# High-Quality Targeted Temperature Management After Cardiac Arrest; Results from the Korean Hypothermia Network Prospective Registry

**DOI:** 10.3390/jcm14165898

**Published:** 2025-08-21

**Authors:** Hyo Jin Bang, Chun Song Youn, Byung Kook Lee, Sang Hoon Oh, Hyo Joon Kim, Ae Kyung Gong, Ji-Sook Lee, Soo Hyun Kim, Kyu Nam Park, In Soo Cho, on behalf of the Korean Hypothermia Network Investigators

**Affiliations:** 1 Department of Emergency Medicine, Seoul St. Mary Hospital, College of Medicine, The Catholic University of Korea, Seoul 06591, Republic of Korea; cleverchoice@catholic.ac.kr (H.J.B.); ohmytweety@catholic.ac.kr (S.H.O.); khjoon0110@catholic.ac.kr (H.J.K.); kyung6069@naver.com (A.K.G.); emsky@catholic.ac.kr (K.N.P.); 2 Department of Emergency Medicine, Chonnam National University Hospital, Chonnam National University of Medical School, 42, Jebong-ro, Donggu, Gwangju 61469, Republic of Korea; bbukkuk@hanmail.net; 3 Department of Emergency Medicine, Eunpyeong St. Mary Hospital, College of Medicine, The Catholic University of Korea, Seoul 03312, Republic of Korea; emksh77@gmail.com; 4 Department of Emergency Medicine, KEPCO Medical Center, 308, Uicheon-ro, Dobong-gu, Seoul 01450, Republic of Korea; mensa@hanmail.net

**Keywords:** cardiac arrest, high-quality TTM, outcome

## Abstract

**Backgrounds:** Most out-of-hospital cardiac arrest (OHCA) survivors are comatose due to hypoxic ischemic brain injury. Targeted temperature management (TTM) is the only evidence-based neuroprotective intervention for this condition; however, the optimal implementation of TTM has yet to be determined. The concept of high-quality TTM has been proposed to improve patient outcomes, but its clinical impact has not been thoroughly evaluated. This study investigates whether adherence to high-quality TTM is associated with improved neurological outcomes and survival among OHCA patients. **Methods**: This retrospective analysis used data from the Korean Hypothermia Network Prospective Registry 1.0, including 1060 adult OHCA patients treated with TTM at 33 °C between 2015 and 2018. High-quality TTM was defined as follows: temperature variability during maintenance within ±1.0 °C, maintenance duration ≥ 24 h, rewarming rate ≤ 0.5 °C/h, and post-TTM fever control (temperature < 38.5 °C). Patients were classified into high- and low-quality TTM groups. The primary outcomes were survival and neurological status (CPC ranging from 1 to 2 indicated a good outcome) 6 months after cardiac arrest (CA). **Results**: Of the 1060 patients, 491 (46.3%) received high-quality TTM. Compared with the low-quality TTM group, the high-quality TTM group had higher rates of survival (44.6% vs. 36.4%, *p* = 0.006). Multivariate analysis revealed that high-quality TTM was independently associated with survival (OR 1.802, 95% CI: 1.171–2.773) and good neurological outcomes (OR 1.748, 95% CI: 1.102–2.770). **Conclusions**: High-quality TTM is associated with improved survival and better neurological outcomes in OHCA patients. Standardizing TTM delivery on the basis of quality metrics may increase its effectiveness in clinical practice.

## 1. Introduction

Most patients who are successfully resuscitated from out-of-hospital cardiac arrest (OHCA) are comatose because of hypoxic ischemic brain injury [1,2]. Currently, targeted temperature management (TTM) is the intervention available to alleviate hypoxic ischemic brain injury in OHCA patients and is thus recommended by international guidelines [3,4]. Although factors such as hyperoxia, hypoxia, hypocapnia, and hypercapnia have been increasingly investigated and acknowledged as critical modulators of neurological outcomes in postcardiac arrest care [5,6], the optimal strategy for implementing TTM itself has not been defined.

TTM is a complex intervention; therefore, there is considerable variance with respect to the devices used to achieve cooling and the protocols, such as induction, target temperature, maintenance, rewarming, and post-TTM fever control [7,8,9]. Several retrospective studies have shown that deviations from the target temperature during TTM, such as overcooling, greater temperature variability during the maintenance phase, and the development of post-TTM fever, may reduce the neuroprotective effects of TTM and ultimately influence neurologic outcomes among OHCA patients [10,11,12,13,14]. Therefore, given the lack of a clearly defined optimal protocol for TTM, stratifying patients with different approaches into TTM treatment arms may be inappropriate. Taccone et al. introduced the concept of high-quality TTM to increase the effectiveness of TTM and standardize its use in future intervention studies [8]. The authors highlighted key components of the TTM protocol, including the timing of initiation, temperature measurement methods, duration of the cooling phase, and duration of the rewarming phase. These four components can be further subdivided to establish a definition of high-quality TTM. However, to date, few studies have investigated whether the application of high-quality TTM leads to actual clinical benefits for patients.

The purpose of this study was to examine the quality of TTM in OHCA patients who were enrolled in a large prospective multicenter registry and to test the hypothesis that high-quality TTM leads to better neurological outcomes than low-quality TTM.

## 2. Materials and Methods

### 2.1. Study Design and Participants

This retrospective observational analysis utilized data from a prospectively maintained, multicenter registry—the Korean Hypothermia Network Prospective Registry 1.0 (KORHN-PRO 1.0)—collected between October 2015 and December 2018 (ClinicalTrials.gov Identifier: NCT02827422). Investigators from 22 participating hospitals entered patient data into a centralized, web-based registry system (http://korhn.mediex.co.kr (accessed on 19 August 2025)). Written informed consent was obtained from patients or their legal surrogates in accordance with the ethical standards of each participating hospital institutional review board, (IRB) including Seoul St. Mary’s hospital (approval code FA-02-02, approval date 21 April 2023) and the principles of the Declaration of Helsinki.

This study included all adult patients (aged ≥18 years) treated with TTM after OHCA. TTM was administered to comatose patients, excluding those with active intracranial hemorrhage, acute stroke, documented limitations in care or do-not-resuscitate orders, a prearrest Glasgow–Pittsburgh Cerebral Performance Category (CPC) of 3 or 4, or a core body temperature of 30 °C upon hospital admission. TTM was performed in accordance with each center’s protocol with respect to the cooling device, target temperature, duration of maintenance, and rewarming speed [9]. Patients were excluded if they received extracorporeal membrane oxygenation (ECMO) during TTM, had a target temperature of 36 °C, or lacked neurological outcome data at the 6-month follow-up.

### 2.2. Data Collection

We collected the following demographic and clinical data from the registry: sex, age, comorbidities (coronary artery disease (CAD), cerebrovascular accident (CVA), hypertension (HTN), diabetes mellitus (DM)), cardiac arrest location, presence of a witness, bystander cardiopulmonary resuscitation (CPR), initial cardiac arrest rhythm (shockable or nonshockable), total anoxic time (time from collapse to return of spontaneous circulation (ROSC), and cause of cardiac arrest.

The following TTM data were prospectively collected in KORHN-PRO 1.0 and used in this study: target temperature, target duration, temperature monitoring site, hourly body temperature from ROSC to 96 h, TTM methods, time from ROSC to the start of TTM, time from the start of TTM to the target temperature, maintenance duration, rewarming duration, controlled normothermia, and duration and maximal temperature after rewarming for 3 days [9].

### 2.3. Definition of High-Quality TTM

**Figure 1:**
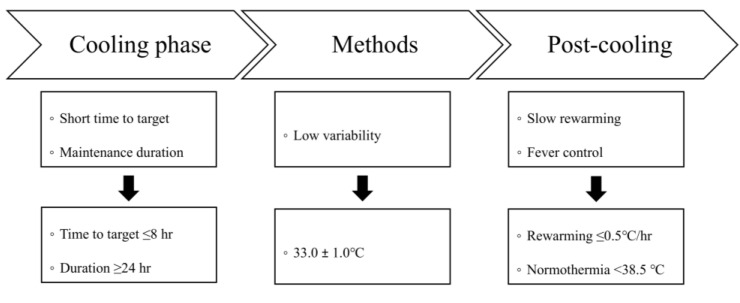
Definition of high-quality TTM.

Because there is no clear definition of high-quality TTM, several detailed TTM-related variables were combined to define high-quality TTM (Figure 1). The following TTM-related variables were considered important by the authors when evaluating TTM quality: target temperature, time from induction to target temperature, maintenance duration, temperature variability during the maintenance phase, rewarming speed, and fever after rewarming. These variables can all be extracted, as body temperature is recorded every hour in KORHN-PRO 1.0. However, the time from induction to the target temperature was not used in the definition of high-quality TTM in this study because of its paradoxical relationship with neurological outcomes in prior investigations [15,16,17]. These retrospective studies indicate that patients with more severe brain injury and impaired thermoregulation may cool more rapidly—not because therapeutic efficiency is greater, but because of passive core heat loss [15,16,17].

In OHCA patients treated with a target temperature of 33 °C, high-quality TTM was defined as having a temperature variability of ±1.0 °C during the maintenance phase, a maintenance duration of at least 24 h, a rewarming rate of ≤0.5 °C/h, and post-TTM fever control by keeping the temperature below 38.5 °C. Patients who did not meet any of the definitions of high-quality TTM were defined as having low-quality TTM.

### 2.4. Outcome Measures

The primary outcomes were survival and neurological status at 6 months after cardiac arrest (CA). Neurological outcomes were assessed by attending physicians or independent neurologists at each hospital and categorized using the CPC as follows: good cerebral performance (CPC 1), moderate cerebral disability (CPC 2), severe cerebral disability (CPC 3), coma or vegetative state (CPC 4), and death (CPC 5). A good neurological outcome was defined as a CPC of 1–2, whereas a poor neurological outcome was classified as a CPC of 3–5. The researcher followed up with surviving discharged patients or their relatives, recommending face-to-face visits or telephone interviews.

### 2.5. Statistical Analysis

All the data are presented as frequencies and percentages (for categorical variables) or as medians with interquartile ranges (IQRs) (for continuous variables). Categorical variables were compared between groups using the chi-square test or Fisher’s exact test. After testing for normality, continuous variables were compared using either Student’s t test (for normally distributed variables) or Wilcoxon’s rank-sum test (for nonnormally distributed variables). To identify independent predictors of survival and good neurological outcomes, all variables with *p* values less than 0.05 in univariate analyses were subsequently included in a multivariate logistic regression model with the enter method. To assess multicollinearity among the independent variables, the variance inflation factor (VIF) and tolerance statistics were calculated using linear regression analysis prior to multivariate logistic regression. All the variables had VIF values less than 2.0 in both outcome models (survival and neurologic outcome), indicating that there was no significant multicollinearity. On the basis of these analyses, odds ratios (ORs) with 95% confidence intervals (CIs) were computed. Statistical analysis was conducted using SPSS version 24.0 (SPSS, Chicago, IL, USA). A *p* value of <0.05 was considered to indicate statistical significance.

## 3. Results

### 3.1. Patients

**Figure 2:**
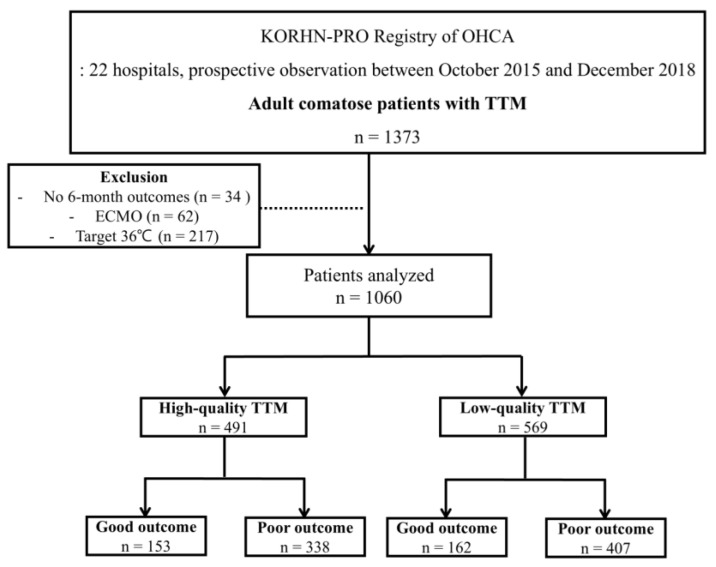
Flow of the study.

The 1373 adult OHCA patients treated with TTM were registered in the KORHN-PRO registry. A total of 313 patients were excluded because they had no 6-month outcomes (34 patients), were treated with ECMO during TTM (62 patients), or had a target temperature of 36 °C. The data of the remaining 1060 patients were analyzed (Figure 2). According to the definition of high-quality TTM, 491 patients (46.3%) were categorized into the high-quality TTM group, and the remaining 569 (53.7%) were categorized into the low-quality TTM group. The demographic and baseline characteristics are described in Table 1. Most of the characteristics, including comorbidities, the presence of a shockable rhythm, and total anoxic time, were not significantly different between the high-quality and low-quality TTM groups. According to the core temperature measurements, more than 98% of patients had TTM. Core temperature was measured mainly with the esophagus, bladder, and rectum. In almost all patients (99.9%), a TTM machine with a temperature feedback function was used.

In the low-quality TTM group, the maintenance time was >24 h in 271 patients (40.0%), the temperature variability was >1 °C in 342 patients (54.6%), the rewarming rate was >0.5 °C in 187 patients (32.9%), and post-TTM fever occurred in 238 patients (41.2%). The percentage of surviving patients at 6 months after CA was greater in the high-quality TTM group than in the low-quality TTM group (44.6% vs. 36.4%, *p* = 0.006). However, the percentage of patients with good neurological outcomes was not significantly different between the groups (31.2% vs. 28.5%, *p* = 0.339).

### 3.2. Quality of TTM According to Neurological Outcomes

The time from induction to the target temperature was longer in the good neurological outcome group than in the poor neurological outcome group. The proportion of patients with a maintenance time of ≥24 h was 82.8% in the good neurological outcome group and 78.6% in the poor neurological outcome group, with no significant difference between the two groups. The percentages of patients with a rewarming rate of ≤0.5 °C/h and post-rewarming fever control below 38.5 °C were significantly greater in the good neurological outcome group (Table 2).

### 3.3. Quality of TTM and Clinical Outcome

Univariate logistic regression analysis included the following variables: male sex, older age, history of HTN and DM, arrest in a public place, witnessed arrest, bystander CPR, shockable initial rhythm, cardiac etiology, total anoxic time, induction time to target temperature, and maintenance time. The warming rate and post-rewarming fever control were associated with survival and good neurologic outcomes (Table 3). According to multivariate logistic regression, high-quality TTM was associated with good neurological outcomes (OR 1.748, 95% CI, 1.102–2.770) and survival (OR 1.802, 95% CI 1.171–2.773) (Table 4). Although the crude comparison of favorable neurological outcomes between high- and low-quality TTM groups did not reach statistical significance (31.2% vs. 28.5%, *p* = 0.339), this unadjusted analysis did not account for baseline imbalances or exposure dilution. As shown in Table 1, a substantial proportion of patients in the low-quality group still received individual quality elements (maintenance ≥ 24 h: 60.0%; variability ≤ ±1.0 °C: 45.4%; rewarming ≤ 0.5 °C/h: 67.1%; post-rewarming fever control < 38.5 °C: 58.2%), which likely attenuated the apparent effect in the crude analysis. To visually assess the prognostic contribution of each quality component, we constructed a supplementary forest plot presenting adjusted odds ratios (aORs) and 95% confidence intervals derived from the multivariable model (Supplementary Figure S1). All confidence intervals crossed 1.0, indicating that no single component could be conclusively identified as more prognostically important in this cohort.

## 4. Discussion

In a large, multicenter, prospective cohort of OHCA patients, we found that low-quality TTM is commonly used among patients treated with a target temperature of 33 °C. Additionally, high-quality TTM, defined as temperature variability during maintenance within ±1.0 °C, maintenance duration ≥ 24 h, a rewarming rate ≤ 0.5 °C/h, and post-TTM fever control (temperature < 38.5 °C), was associated with survival and good neurological outcomes at 6 months after CA.

Our study has several strengths. First, KORHN-PRO 1.0 is a large-scale, multicenter, prospective registry that accurately reflects the real-world clinical situation [9]. Second, patients with mixed etiologies of CA not limited to cardiac causes were included, thus increasing the generalizability of this study. Finally, this is the first study to demonstrate an association between high-quality TTM and patient outcomes. We hypothesized that high-quality TTM leads to better neurological outcomes than low-quality TTM does. The entire TTM process was examined herein to assess its association with outcomes rather than addressing one phase of TTM.

There may be some controversy regarding the definition of high-quality TTM used in this study, especially the exclusion of “time from induction to target temperature.” Several experimental studies have shown that the faster the target temperature is reached after ROSC, the better the neurological outcome [18,19]. However, several retrospective clinical studies have reported a paradoxical association in which shorter induction times are linked to poor neurological outcomes [15,16,17]. This counterintuitive finding may be attributed to confounding indications, in which patients with more severe brain injury tend to cool more rapidly through passive mechanisms, likely due to impaired thermoregulatory function or diminished endogenous heat production [15,16,17]. Therefore, TTM must be initiated quickly after ROSC, and the target temperature must be reached quickly after TTM initiation. Moreover, the use of intraarrest TTM with a transnasal device has shown some beneficial effects in OHCA patients with an initial shockable rhythm [20,21]. Some researchers have argued that the time from induction to the target temperature should be within 4 or 8 h [7,22]. In prospective studies, the time from induction to the target temperature variable should be included in the definition of high-quality TTM, and clinicians should make every effort to reach the target temperature within the specified time. However, this was a retrospective study; therefore, a paradoxical relationship may exist, such that patients with a long induction time have good neurological outcomes [15,16,17]. Therefore, the time from induction to the target temperature was not used in the definition of high-quality TTM herein. However, this variable should be included in future prospective studies.

The number of cases treated with a target temperature of 36 °C after CA has increased worldwide since the publication of the TTM trial [23]. Additionally, actively preventing fever by targeting a temperature < 37.5 °C for patients who remain comatose after CA was recently recommended by the 2024 International Liaison Committee on Resuscitation (ILCOR) [24]. Most patients included in the KORHN-PRO 1.0 registry were treated with a target temperature of 33 °C or 36 °C. The reason why the target temperature of 33 °C was exclusively included in this study is that the current study aimed to examine whether high-quality TTM improves neurological outcomes, not whether 33 °C is a more effective temperature than 36 °C is. The exclusive inclusion of patients with a target temperature of 33 °C was an ideal model to achieve this aim.

Despite the lack of clinical evidence, it is common for the temperature to deviate from the target temperature during the TTM maintenance period, and many experts recommend reducing temperature variability through appropriate sedation, the use of NMB, and the use of autofeedback devices [25,26]. This is because overcooling or overshooting beyond the target temperature range can affect patient outcomes. Although temperature variability has been reduced with the introduction of autofeedback devices, a well-designed RCT reported that overcooling occurred in 13% of all patients and that overshoot occurred in 26%, indicating that this phenomenon still occurs quite frequently [22].

It is recommended that rewarming rates be as slow as 0.5 °C/h or less. To achieve this rate, active controlled rewarming (rather than passive rewarming) should be used with specific TTM devices, as passive rewarming can result in unpredictable rates. Hifumi et al. reported that a longer rewarming duration was an independent predictor of favorable neurological outcomes in OHCA patients who received TTM [27]. The development of fever after TTM is not uncommon. Several retrospective studies have shown that fever after TTM is associated with unfavorable outcomes and that fever should generally be prevented for up to 72 h after rewarming [11,12]. However, a recent RCT revealed that active device-based fever prevention for 36 or 72 h after CA did not result in significantly different percentages of patients dying or experiencing severe disability or coma [28].

With the recent development of TTM equipment, the implementation of TTM has become easier. Additionally, it has become easier to control each stage of TTM, such as induction, maintenance, rewarming, and post-rewarming fever control. Therefore, RCTs have examined the relationship between the control of each stage and clinical outcomes; these trials have yielded mixed results [20,21,28,29]. However, the current study is significant in that it does not address only one stage of the TTM but rather treats the entire TTM process as a single variable and demonstrates its association with patient outcomes. Additionally, we demonstrated that patient outcomes can be improved if the quality of the entire TTM process is well maintained. Although univariate analysis suggested that some individual components of high-quality TTM, such as the rewarming rate and prevention of post-TTM fever, were more strongly associated with favorable outcomes, these associations were not statistically significant after adjustment for potential confounders when all components were included in the multivariable model regardless of their univariate significance. As shown in the supplementary forest plot, all confidence intervals crossed 1.0, indicating that no single component could be definitively prioritized in terms of prognostic importance in this cohort.

The lack of a significant crude difference between the high- and low-quality TTM groups (31.2% vs. 28.5%, *p* = 0.339) likely reflects both baseline imbalances and exposure dilution. As shown in Table 1, many patients in the low-quality group still received individual quality components (maintenance ≥ 24 h: 60.0%; variability ≤ ±1.0 °C: 45.4%; rewarming ≤ 0.5 °C/h: 67.1%; post-rewarming fever control < 38.5 °C: 58.2%), which attenuated the apparent effect in unadjusted analyses. Moreover, crude comparisons did not account for differences in prognostic factors such as induction time to target temperature and core temperature monitoring. After adjusting for these and other covariates, high-quality TTM emerged as an independent predictor of good neurological outcomes. These findings suggest that the benefits of TTM are likely attributable to adherence to multiple quality measures rather than to any single element and should be interpreted as exploratory. There are concerns that the importance of TTM may decrease, as the ILCOR recommends active fever prevention by maintaining body temperature below <37.5 °C after CA [24]. Although this study does not present a standard for high-quality TTM, further research is needed to define high-quality TTM across multiple phases, including achieving and maintaining the target temperature, rewarming, and post-TTM fever prevention, and should be considered in future large-scale RCTs.

This study has several limitations. First, although the KORHN-PRO data were prospectively collected from TTM patients, the nature of this retrospective analysis led to bias, including selection bias, information bias, and residual confounding. For example, treatment decisions and documentation practices may have varied across participating centers, and not all relevant covariates could be adjusted for, potentially affecting the observed associations. Second, the time from induction to the target temperature was not used in the definition of high-quality TTM in this study. This variable should be considered in future prospective studies. Third, the results cannot be generalized to patients treated with other target temperatures because only patients treated with a target temperature of 33 °C were included. Other temperature cut-offs, such as 36 °C, were not evaluated in this study, and further research is warranted to determine whether the findings apply across different TTM protocols. Fourth, the generalization of TTM may be limited by resource constraints, such as access to closed-loop cooling systems or comprehensive post-resuscitation care infrastructure. In the Korean healthcare system, the cost per patient undergoing TTM ranges from approximately USD 1300 to 1800, depending on insurance coverage. These costs pertain solely to the TTM procedure itself and exclude additional expenses such as ICU stay, patient monitoring, and other concurrent treatments. These financial and infrastructural limitations may affect the widespread implementation of TTM, particularly in resource-limited settings. Finally, different sites were used to measure the core temperature, which may have introduced measurement bias. Although core temperature was measured at most sites, variability in measurement locations could have led to inconsistent temperature readings. Esophageal temperature correlates most closely with pulmonary artery temperature and responds more rapidly to changes, whereas bladder and rectal temperatures may demonstrate delayed thermal responses, especially during the induction phase of TTM [30,31,32].

## 5. Conclusions

In this multicenter observational study, the application of high-quality TTM among comatose OHCA survivors was associated with significantly improved survival and neurological outcomes at 6 months. These findings support the clinical importance of standardized TTM protocols that emphasize key quality indicators, such as controlled temperature variability, adequate maintenance duration, slow rewarming, and effective post-TTM fever management. Incorporating the concept of high-quality TTM into routine clinical practice and future research may help optimize neuroprotection and lead to favorable outcomes among OHCA patients.

## Figures and Tables

**Table 1:** Demographic and baseline characteristics according to quality of TTM.

	Total (N = 1060)	High-Quality TTM (N = 491)	Low-Quality TTM (N = 569)	*p*-Value
Male (*n*)	751 (10.8%)	334 (68.0%)	417 (73.3%)	0.060
Age (year, IQRs)	58.0 (47.0–69.0)	59.0 (48.0–69.0)	57.0 (46.0–68.0)	0.937
BMI (kg/m^2^, IQRs)	23.4 (21.0–25.7)	23.1 (20.8–25.8)	23.7 (21.5–25.9)	0.267
Comorbidity (*n*)				
History of CAD	125 (11.8%)	54 (11.0%)	71 (12.5%)	0.456
History of CVA	58 (5.5%)	30 (6.1%)	28 (4.9%)	0.396
History of HTN	389 (36.7%)	187 (38.1%)	202 (35.5%)	0.384
History of DM	269 (25.4%)	137 (27.9%)	132 (23.2%)	0.079
Cardiac arrest at public place (*n*)	500 (47.2%)	244 (49.7%)	256 (45.0%)	0.126
Witnessed arrest (*n*)	755 (71.2%)	348 (70.9%)	407 (71.5%)	0.815
Bystander CPR (*n*)	645 (60.8%)	311 (68.3%)	334 (58.7%)	0.123
Shockable initial rhythm (*n*)	355 (33.5%)	156 (31.8%)	199 (35.0%)	0.271
Total anoxic time (min, IQRs)	25.0 (15.0–38.0)	25.0 (15.0–38.0)	25.0 (15.0–38.0)	0.522
Cardiac etiology (*n*)	654 (61.7%)	294 (59.9%)	360 (63.3%)	0.257
Core temperature measurement (*n*)	1040 (98.1%)	487 (99.2%)	553 (97.2%)	0.017
Temperature feedback method (*n*)	1058 (99.8%)	490 (99.8%)	568 (99.8%)	0.917
Induction time to target temperature ≤ 8 h (*n*)	719 (67.8%)	344 (70.1%)	375 (65.9%)	0.149
Induction time to target temperature (h, IQRs)	2.0 (1.0–4.0)	2.0 (1.0–3.0)	3.0 (1.0–5.0)	0.004
Maintaining time ≥ 24 h (*n*)	789 (79.9%)	491 (100%)	298 (60.0%)	<0.001
Maintaining time (h, IQRs)	28.0 (26.0–30.0)	28.0 (27.0–29.0)	27.0 (21.0–30.0)	<0.001
Variability during TTM ± 1.0 °C (*n*)	718 (72.5%)	491 (100%)	227 (45.4%)	<0.001
Variability during TTM (°C, IQRs)	1.4 (1.1–1.8)	1.2 (1.0–1.4)	1.5 (0.8–2.1)	<0.001
Rewarming rate ≤ 0.5 °C/h (*n*)	873 (82.4%)	491 (100%)	382 (67.1%)	<0.001
Rewarming rate (°C/h, IQRs)	0.25 (0.21–0.30)	0.25 (0.21–0.27)	0.26 (0.20–0.33)	<0.001
Post-rewarming fever control < 38.5 °C (*n*)	822 (77.5%)	491 (100%)	331 (58.2%)	<0.001
Survival at 6 months (*n*)	426 (40.2%)	219 (44.6%)	207 (36.4%)	0.006
Good outcome at 6 months (*n*)	315 (29.7%)	153 (31.2%)	162 (28.5%)	0.339

Data are presented as *n* (%) for categorical variables and as medians (interquartile ranges, IQRs) for continuous variables. Abbreviations: BMI, body mass index; CAD, coronary artery disease; CVA, cerebrovascular accident; HTN, hypertension; DM, diabetes mellitus; CPR, cardiopulmonary resuscitation; TTM, targeted temperature management.

**Table 2:** TTM-related variables according to neurological outcomes.

	Good Outcome (N = 315)	Poor Outcome (N = 745)	*p*-Value
Core temperature measurement (*n*)	309 (98.1%)	731 (98.1%)	0.978
Temperature feedback method (*n*)	315 (100%)	743 (99.7%)	0.357
Induction time to target temperature ≤ 8 h (*n*)	162 (51.4%)	557 (74.8%)	<0.001
Induction time to target temperature (h, IQRs)	3.0 (2.0–5.3)	2.0 (1.0–3.0)	<0.001
Maintaining time ≥ 24 h (*n*)	250 (82.8%)	539 (78.6%)	0.128
Maintaining time (h, IQRs)	28.0 (26.0–29.3)	28.0 (26.0–30.0)	0.324
Variability during TTM ± 1.0 °C (*n*)	218 (72.2%)	500 (72.6%)	0.901
Variability during TTM (°C, IQRs)	1.4 (1.1–1.7)	1.3 (1.1–1.8)	0.064
Rewarming rate ≤ 0.5 °C/h (*n*)	281 (89.2%)	592 (79.5%)	<0.001
Rewarming rate (°C/h, IQRs)	0.26 (0.22–0.33)	0.25 (0.20–0.29)	<0.001
Post-rewarming fever control (<38.5 °C, *n*)	256 (82.1%)	566 (89.6%)	0.001

Data are presented as *n* (%) for categorical variables and as medians (interquartile ranges, IQRs) for continuous variables.

**Table 3:** Univariate logistic regression analysis for survival and good neurological outcome at 6 months after CA.

	Survival			Good Outcome		
	OR	95% C.I.	*p*-Value	OR	95% C.I.	*p*-Value
Male Sex	1.394	1.058–1.836	0.018	1.575	1.161–2.136	0.004
Age	1.026	1.017–1.034	<0.001	1.035	1.026–1.044	<0.001
BMI	0.995	0.965–1.025	0.730	0.997	0.966–1.030	0.871
History of CAD	1.194	0.820–1.741	0.355	1.385	0.936–2.050	0.103
History of CVA	0.977	0.569–1.678	0.932	0.896	0.496–1.618	0.715
History of HTN	0.625	0.482–0.811	<0.001	0.597	0.449–0.793	<0.001
History of DM	0.508	0.377–0.685	<0.001	0.453	0.322–0.637	<0.001
Cardiac arrest at public place	1.633	1.275–2.092	<0.001	1.586	1.217–2.068	<0.001
Witnessed arrest	3.068	2.264–4.158	<0.001	3.321	2.335–4.724	<0.001
Bystander CPR	1.299	1.008–1.675	0.043	1.37	1.041–1.804	0.025
Shockable initial rhythm	7.793	5.845–10.390	<0.001	15.299	11.108–21.073	<0.001
Total anoxic time	1.045	1.036–1.055	<0.001	1.056	1.044–1.067	<0.001
Cardiac etiology	3.946	2.975–5.233	<0.001	7.647	5.252–11.133	<0.001
Induction time to target temperature	0.820	0.778–0.865	<0.001	0.814	0.773–0.858	<0.001
Maintaining time	1.610	1.160–2.235	0.004	1.311	0.924–1.861	0.129
Variability during TTM	1.146	0.862–1.523	0.349	0.981	0.725–1.327	0.901
Rewarming rate	3.258	2.220–4.782	<0.001	2.136	1.435–3.180	<0.001
Post-rewarming fever control	0.518	0.352–0.762	<0.001	0.533	0.363–0.784	0.001
High-quality TTM	1.408	1.100–1.802	0.007	1.137	0.873–1.481	0.339

Abbreviations: BMI, body mass index; CAD, coronary artery disease; CVA, cerebrovascular accident; HTN, hypertension; DM, diabetes mellitus; CPR, cardiopulmonary resuscitation; TTM, targeted temperature management.

**Table 4:** Multivariate logistic regression analysis for survival and good neurological outcomes.

	Survival			Good Outcome		
	OR	95% C.I.	*p*-Value	OR	95% C.I.	*p*-Value
High-quality TTM	1.802	1.171–2.773	0.007	1.748	1.102–2.770	0.018

## Data Availability

The raw data supporting the conclusions of this article will be made available by the authors on request.

## Supplementary Materials

The following supporting information can be downloaded at: https://www.mdpi.com/article/10.3390/jcm14165898/s1, Figure S1: Adjusted odds ratios (aORs) and 95% confidence intervals (CIs) for individual components of high-quality targeted temperature management (TTM) in relation to favorable neurological outcome; Table S1: Lists of the principle investigator, IRB board name, approval number, approval date from each participating site.
