# Supplementary material for: High-Quality Targeted Temperature Management After Cardiac Arrest; Results from the Korean Hypothermia Network Prospective Registry
Source: J Clin Med. 2025 Aug 21;14(16):5898. doi: 10.3390/jcm14165898 (PMC12387557; doi:10.3390/jcm14165898)

Supplementary Table S1. Lists of the Principle investigator, IRB board name, approval number, approval date from each participating site entitled "A prospective, multicenter registry for evaluating real-world effectiveness, safety and outcome-associated factors of post-cardiac arrest care including targeted temperature management after out-of-hospital cardiac arrest in Korea" in the KORHN-PRO 1.0

Principle investigator	Sites	IRB board name	Approval number	Approval date	Study title
Kyu Nam Park<br>Won Jung Jeong<br>Seung Pill Choi<br>Joo Suk Oh	Seoul St. Mary's Hospital<br>St. Vincent's Hospital<br>Yeouido St. Mary's Hospital<br>Uijeongbu St. Mary's Hospital	Catholic Medical Center Central IRB	XC15OIMI0081	October 7, 2015	A prospective, multicenter registry for evaluating real-world effectiveness, safety and outcome-associated factors of post-cardiac arrest care including targeted temperature management after out-of-hospital cardiac arrest in Korea
Je Sung You	Gangnam Severance Hospital	Institutional Review Board of the Yonsei University Health System	3-2015-0247	November 10, 2015	
Jong-Seok Lee	Kyung Hee University Medical Center	Kyung Hee University Hospital Institutional Review Board	KMC IRB 1529-02	November 16, 2015	
Su Jin Kim	Korea University Anam Hospital	Korea University Medical Center Institutional Review Board	2015AN0306	November 2, 2015	
Tae Chang Jang	Daegu Catholic University Medical Center	Daegu Catholic University Medical Center IRB	CR-15-100-L	December 2, 2015	
Jae Hoon Lee	Dong-A University Hospital	Dong-A University Hospital IRB Committee	DAUHIRB-16-079	April 27, 2016	
Min Seob Sim	Samsung Medical Center	Samsung Medical Center Institutional Review Board	SMC 2016-03-104	April 1, 2016	
Won Young Kim	Asan Medical Center	The Institutional Review Board of Asan Medical Center	No. 2015-1052	September 23, 2015	
Jonghwan Shin	Seoul Metropolitan Government Seoul National University Boramae Medical Center	Seoul National University Boramae Medical Center Institutional Review Board	16-2015-109	August 26, 2015	

continued..

Principle investigator	Sites	IRB board name	Approval number	Approval date	Study title
Yoo Seok Park	Severance Hospital	Yonsei University Health System, Severance Hospital, Institutional Review Board	4-2015-0690	September 9, 2015	A prospective, multicenter registry for evaluating real-world effectiveness, safety and outcome-associated factors of post-cardiac arrest care including targeted temperature management after out-of-hospital cardiac arrest in Korea
Hyung Jun Moon	Soonchunhyang University Cheonan Hospital	The Soonchunhyang University Institutional Review Board	2015-07-024	September 24, 2015	
Minjung Kathy Chae	Ajou University Medical Center	Ajou University Hospital IRB	AJOUIRB-OBS-2015-373	November 20, 2015	
Kyoung-Chul Cha	Wonju Severance Christian Hospital	Institutional Review Board of Wonju Severance Christian Hospital	CR315043	December 29, 2015	
Yoon Hee Choi	Ewha Womans University Mokdong Hospital	Ewha Womans University Mokdong Hospital Institutional Review Board	EUMC 2015-08-021-001	September 21, 2015	
Byung Kook Lee	Chonnam National University Hospital	Chonnam National University Hospital IRB	CNUH-2015-164	August 18, 2015	
Dong Hoon Lee	Chung-Ang University Hospital	Chung-Ang University Hospital Institutional Review Board	C2015162(1620)	December 4, 2015	
Yong Hwan Kim	Samsung Changwon Hospital	Samsung Changwon Hospital Institutional Review Board	SCMC 2015-10-05	October 27, 2015	
In Soo Cho	Hanil General Hospital	General Hospital Institutional Review Board	HIRB-2015-012	January 8, 2016	

Supplementary Figure S1 Adjusted odds ratios (aORs) and 95% confidence intervals (CIs) for individual components of high-quality targeted temperature management (TTM) in relation to favorable neurological outcome. Estimates were obtained from a multivariable logistic regression model that included all quality components regardless of their univariate p-values, adjusted for predefined covariates. The vertical dashed line represents an aOR of 1.0. All CIs cross 1.0, indicating that no single component demonstrated a statistically significant independent association with outcome. Component-level results should be interpreted as exploratory.

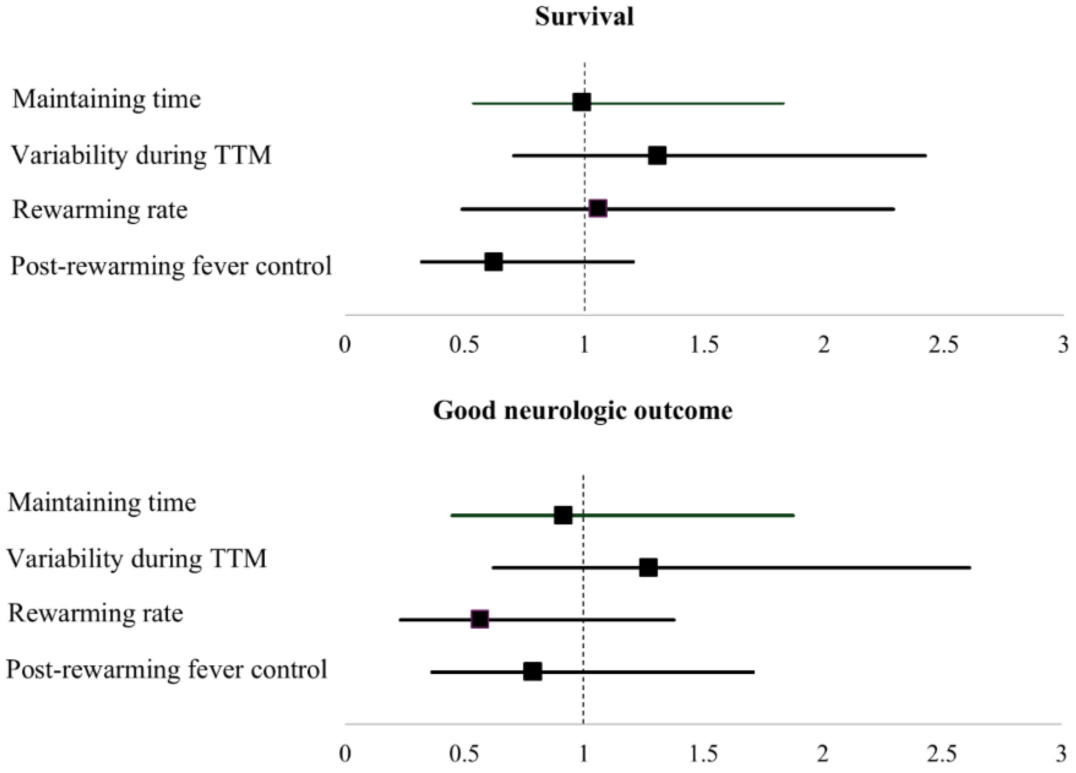

Supplement: Supplementary file 1 [file jcm-14-05898-s001.zip › jcm-3776990_SupplementaryMaterials.pdf]
