# Supplementary figures and images for: High-Quality Targeted Temperature Management After Cardiac Arrest; Results from the Korean Hypothermia Network Prospective Registry
Source: J Clin Med. 2025 Aug 21;14(16):5898. doi: 10.3390/jcm14165898 (PMC12387557; doi:10.3390/jcm14165898)

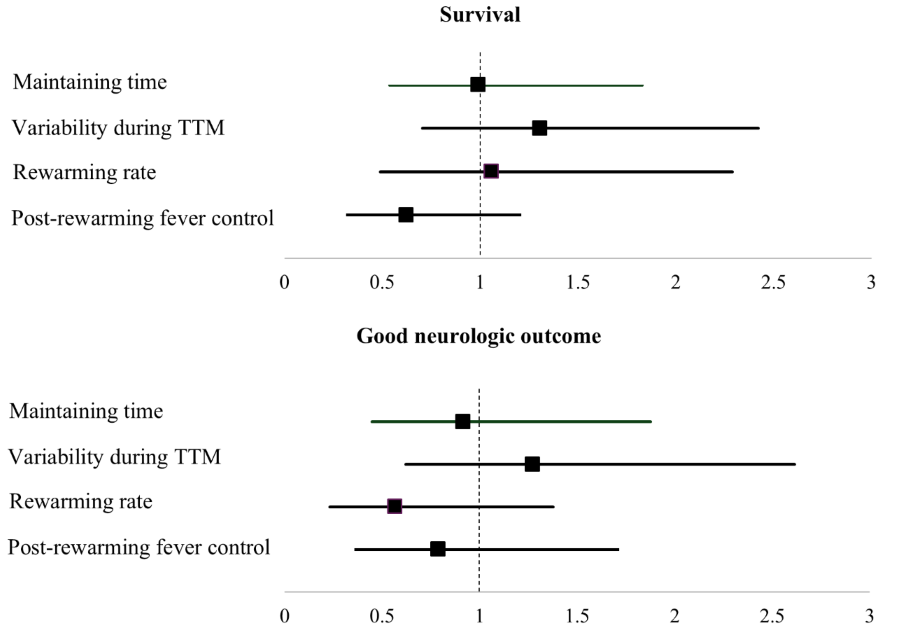

Supplement: Supplementary file 1 [file jcm-14-05898-s001.zip › jcm-3776990_Supplementary Figure S1.tif]
